# Supplementary material for: Are carbon emissions trading and green financial instruments synergistic? -Comprehensive quantitative research based on content analysis
Source: PLoS One. 2024 Mar 7;19(3):e0298601. doi: 10.1371/journal.pone.0298601 (PMC10919601; doi:10.1371/journal.pone.0298601)
Supplement: S1 Data — (ZIP) [file pone.0298601.s001.zip › Data/地级市Z_F工作报告.zip!/╣π╢1⁄2/╔╟╬▓╩╨/└┤╫╘╣1⁄2╓┌║┼í╛┬φ┐╦╩2╛▌═°í┐_2009.docx]

各位代表：

　　我代表市人民政府向大会作政府工作报告，请予审议，并请政协各位委员和其他列席人员提出意见。

2008年工作回顾

　　2008年是我国一个很不寻常的年头，我市也从中经受住了重大考验。一年来，在省委、省政府和市委的正确领导下，在市人大、市政协的监督、支持下，市政府高举中国特色社会主义伟大旗帜，以邓小平理论和"三个代表"重要思想为指导，全面贯彻落实科学发展观，深入贯彻落实党的十七大、十七届三中全会、省十次党代会精神，紧紧围绕市五次党代会和市五届人大一次会议提出的打造"三大基地"、建设"三大经济带"、实施"八大工程"的总体要求，沉着应对国际金融危机对我市经济社会发展的严峻影响，一手抓经济发展，一手抓社会稳定，促进了国民经济持续、快速、健康发展，顺利完成了市五届人大三次会议确定的各项任务，经济社会建设取得了难得的新成就，"活力汕尾、人文汕尾、和谐汕尾、清新汕尾"建设取得了新进展。概括起来，主要体现在十个方面：

　　1、综合实力不断增强，人民生活不断改善。预计全年完成地区生产总值355亿元，比增16.2%。工业总产值570亿元，比增24.0%。全社会固定资产投资207.5亿元，比增18.0%。合同吸收外资4.14亿美元，比增3.0%，实际吸收外商直接投资2.56亿美元，比增26.0%。社会消费品零售总额232亿元，比增24.0%。农业总产值106亿元，比增6.0%。三次产业结构进一步优化，由2007年的18.5∶46.5∶35调整为18∶47∶35，全年一、二、三产业对经济增长的贡献率分别为6.2%、63.7%和30.1%。城镇居民人均可支配收入11060元，人均消费支出8247元，分别比增15.6%和6.8%。农民人均纯收入4790元，比增12.1%。万元GDP能耗控制在0.546吨标准煤以内。

　　2、工业规模不断壮大，基地经济初具规模。预计全年完成规模以上工业总产值269亿元，比增34.0%。"三大基地"建设扎实推进，以汕尾电厂、陆丰核电等为引擎的电力能源产业基地初显雏形，以信利、德昌电子企业为龙头的电子信息产业基地初具规模，以中石油华城石化项目为新起点的石化产业基地建设开局良好。以专业镇为载体的毛织、服装、珠宝、金银首饰、制鞋等加工业已形成产业集群。"双转移"工作顺利推进，工业园区建设步伐加快，深圳（汕尾）产业转移工业园动工建设，一批产业转移园区、经济开发区、工业区成为我市工业经济发展的重要载体。

　　3、投资规模持续扩大，重点项目进展顺利。市22项重点建设项目稳步推进，预计全年完成投资25.7亿元。厦深铁路汕尾段、陆丰核电进厂道路、电网建设、市区金湖路、香洲东路、汕尾市技工学校扩建工程、汕尾市101工程、省道S241线汕尾至遮浪段扩建、公平灌区总干渠加固改造工程等项目进展顺利。陆丰核电站、汕尾电厂3#、4#机组、华润海丰电厂、厦深铁路汕尾段、龙川至汕尾铁路等项目列入省新十项工程。投资势头不减，全市新登记企业377家。

　　4、"三农"工作扎实推进，绿色、蓝色经济带生机盎然。预计全年完成粮食种植面积153.15万亩，总产48.85万吨，分别比增7.9%、12.5%；全年渔业总产量53.75万吨，比增2.2%。粮食四项考评工作全面达标。一批粮食高产示范基地、优质无公害蔬菜生产基地等特色种植业基地建设步伐加快。农业产业化经营发展加快。全市已有农业龙头企业39家，其中国泰食品有限公司被国家八部委授予"农业产业化国家重点龙头企业"称号，填补了我市的空白。新农村建设取得新成效。扶贫工作稳步推进，强农惠农政策进一步落实，林业生态建设取得新成绩。

　　5、财税工作成效突出，金融形势保持稳定。全年完成财政一般预算收入15.75亿元，比2007年增加3.59亿元，比增29.6%，增幅列全省第三。其中，国税部门完成地方收入2.5亿元，比增25.2%；地税部门完成地方收入6.0亿元，比增20.8%。财政收支基本平衡。金融形势稳定，预计2008年末金融机构各项存款余额225亿元，比年初增长17.0%，各项贷款余额77亿元，比年初增长8.0%。

　　6、利用外资平稳增长，对外贸易结构优化。由于国际金融危机、世界经济增长放缓以及国家经济政策调整等原因，我市外源经济发展也受到一些影响，但总体上仍然保持平稳发展势头。预计全年合同吸收外资、实际利用外商直接投资分别比增3.0%、26.0%；外贸出口总值11亿美元，基本与2007年持平。其中，一般贸易出口1.5亿美元，比增66.0%，占全市出口的14.0%,比2007年提升6个百分点。与港澳台、珠三角等地区的经济协作进一步加强。

　　7、商贸旅游日显繁荣，市场秩序持续规范。高度重视市场安全，审慎应对"问题奶粉"等食品安全问题，没有发生食品安全事故。预计全年完成社会消费品零售总额232亿元，比增24.0%。个体私营经济进一步发展。全市现有个体工商户5.4万户，私营企业2957家，实现民营经济增加值210亿元，占全市生产总值59.2%。我市"大哥大"、"百斯盾"两个商标被评为"中国驰名商标"，实现零的突破。全市著名商标累计达到11个。旅游配套设施建设扎实推进，旅游发展环境不断优化，旅游品牌塑造得到加强，全市旅游项目完成投资4亿元。预计全年旅游总收入19.03亿元，旅游创汇550万美元，分别比增11.7%、5.5%。

　　8、积极打造城市品牌，投资环境明显提升。重新审视城市自然禀赋和区位条件，适时提出"发展海岸经济、崛起蓝色汕尾"战略，投资吸引力明显提升。城市交通与基础设施建设不断完善。香洲东路首期工程、腾飞路东段等市区主要道路已建成通车；巴黎半岛酒店已建成开业；市体育馆已投入使用；集市区防洪、市政道路、城市景观为一体的环品清湖综合开发项目已完成防洪（潮）堤围建设8公里，城市空间进一步拓展。城市规划建设管理得到加强，市容市貌明显改善。成功举办了中国（汕尾）老区发展论坛暨汕尾建市20周年经贸活动、汕尾海岸经济发展论坛、深圳（汕尾）产业转移招商推介会等大型活动，积极参加首届侨博会、山洽会、中小企业博览会，取得合作意向、协议、合同共900多亿元，汕尾的对外形象和影响力明显提升。环保工作得到加强，污染减排及污染源普查工作扎实推进，全市生态环境总体良好。

　　9、社会事业不断发展，文化建设逐步繁荣。义务教育得到巩固提高，全面实现了免费义务教育，适龄儿童入学率达到98.7%，初中适龄儿童、青少年入学率达98.9%，初中毕业生升学率提高6个百分点。普及高中阶段教育稳步推进，高中毛入学率提高6个百分点，2008年高考入围人数突破万人大关。文化工程建设亮点迭出，继2008年初获得"中国民间文化艺术之乡"荣誉称号之后，又有麒麟舞、河田高景入选第二批国家非物质文化遗产，艺术品种参加国家和省级大赛取得"两金三银"的好成绩。以"欢乐广场、唱响汕尾"为主题的群众性广场文化活动蓬勃兴起，文化出版市场整治得到加强。广播电视工作取得新进展。"农家书屋工程"建设扎实推进。体育场地设施进一步改善，竞技体育取得新成绩，参加粤东四市龙舟赛夺得"头标"。城乡公共医疗卫生服务进一步改善。城乡人口与计划生育工作得到加强。社会救助、扶贫、救灾救济、残疾人事业不断推进。殡葬管理得到强化。国防工作进一步加强。军政、军民团结更加紧密，首次荣获"全省双拥模范城"称号。妇女儿童发展规划进一步落实，未成年人和妇女权益得到维护。统计、审计、人防、气象、防震、台务、物价、司法、港澳、老龄、民族宗教、外事侨务、档案修志等工作取得新成绩。

　　10、民主法制建设逐步加强，"和谐汕尾"建设全面推进。自觉接受人大监督、政协民主监督、新闻舆论监督和公众监督，积极办理人大代表议案、建议和政协委员提案，政府工作的法制化水平不断提高。基层民主建设和村民自治工作得到加强，基本完成全市村（居）委会换届选举工作。社会管理和维稳工作进一步加强。社会治安综合治理目标管理责任制得到落实，"平安工程"建设全面推进。

　　各项严打专项整治活动深入开展，"两抢一盗"、凶杀贩毒等严重违法犯罪活动得到有效遏制。走私贩私活动处于低发可控状态。安全生产"三大体系"得到落实，安全生产四项指标全面下降，安全生产形势持续稳定。公共安全突发事件预防应急管理工作得到强化。县委书记大接访活动成效明显，信访维稳工作机制不断完善。矛盾排查调处工作不断加强，民生问题得到妥善解决，奥运会等重要时段全市平稳安定。2008年全市新增城镇就业4万人，转移农村劳动力就业3.8万人。城镇登记失业率控制在3.0%以内。养老、失业、工伤等社会保险覆盖面继续扩大。全市征收五项社保费4.5亿元，比增29.0%,企业离退休人员养老金和各项社保待遇按时足额发放。新型农村合作医疗覆盖面100%，参合率达99.1%。全面启动城镇居民基本医疗保险。双拥工作大力推进，困难企业军转干部生活困难补贴和驻汕市区部队生活补贴得到提高，城镇退役士兵得到妥善安置，重点优抚对象的基本生活得到保障。低保覆盖面继续扩大，补助标准不断提高。发放城乡低保保障金5904万元，享受低保救助对象3.46万户，8.3万人。涉及人民群众切身利益的住房、饮水、打官司、子女读书等问题得到较好解决。

　　与此同时，市政府切实加强自身建设，认真贯彻落实《行政许可法》、《全面推进依法行政实施纲要》等法律法规，行政效能监察不断强化，"法治政府"建设不断加强。行政执法责任制、廉政建设责任制进一步落实，各项规章制度进一步健全和完善，廉洁从政教育和警示教育初见成效。深入开展"行风热线"、行风评议和万众评公务活动，损害群众利益的不正之风得到遏制，机关作风明显转变，办事效率和服务水平进一步提高。

　　各位代表，2008年取得的成绩来自不易，是在省委、省政府和市委的正确领导下，在市人大及其常委会、市政协的大力支持下，全市人民团结奋斗、共克时艰的结果。在此，我谨代表市人民政府，向全市广大工人、农民、知识分子、干部职工，向驻汕人民解放军、武警官兵和各民主党派、人民团体、社会各界人士，表示崇高的敬意！向所有关心支持我市发展的港澳同胞、台湾同胞、海外侨胞以及国际友人表示衷心的感谢！

　　回顾2008年的工作,我们有很多有益的启示：一是必须以科学发展观为指导，牢牢把握科学发展、跨越发展主题，加快转变经济发展方式，不断优化发展环境，打造新的发展平台。二是必须不断解放思想，坚持改革开放，与时俱进，以思想大解放促进经济社会大发展。三是必须立足大视野、规划大经济、谋划大发展，突出抓好招商引资工作，大力推进重点项目建设，努力推进经济新跨越。四是必须加强对经济工作的领导，强化责任，奋发有为，真抓实干。五是必须以人为本，高度关注民生，维护民利，让发展成果惠及全市人民。

　　在肯定成绩的同时，我们也清醒地看到，我市经济社会发展仍存在一些问题和困难，主要是：经济总体规模小，与发达地区的差距较大，抗击风险能力较弱；受世界金融危机冲击，外贸进出口等重要经济指标增长缓慢；转变经济发展方式、调整经济结构的任务还比较艰巨；基础设施建设滞后，部分重点项目推进缓慢；自主创新能力不高；县域经济发展不均衡，农民渔民人均收入仍然偏低；财政调控能力不强，社会发展基础薄弱；教育、卫生、殡改、人口计生、社会保障、社会治安等方面工作压力较大；软环境建设滞后，思想观念、社会风气、社会环境、政务环境和人才建设与发展的要求还不相适应。对此，我们必须高度重视，切实采取措施认真解决。

2009年工作安排

　　2009年是新中国成立60周年，是推进"十一五"规划、实施"砍尾"行动的关键之年，也是我市应对世界金融危机、共克时艰的考验之年。做好全年各项工作，意义重大，责任重大。纵观国际国内形势，我们既面临严峻的挑战，也迎来新的发展机遇。一方面，我们要充分估计到，世界金融危机，全球经济、国内经济放缓必将对我市加快发展造成一定的冲击和影响。另一方面，我们更要看到，为应对全球金融危机，国家实行积极的财政政策和适度宽松的货币政策，出台了进一步扩大内需、促进经济增长的十项措施，并将投入4万亿元的配套资金。中央经济工作会议把扩内需、保增长、促发展作为今年的首要任务。省也相应出台了十六项措施，2009年投资1000亿元，将带动社会投资1.3万亿元，推动经济平稳较快发展。这些都为我市加快发展提供了一系列利好因素。从我市现实看，经过建市20年的奋斗，经济社会发展的基础日渐厚实，自我发展的能力逐步增强。随着我市列入省新十项工程等一批重大项目，以及深圳（汕尾）产业转移工业园建设的加快推进，资源优势、区位优势和政策优势将进一步得以发挥，发展动力将日益增强。特别是当前全市上下实施"砍尾"行动、推进科学发展、跨越发展的热情高涨，为我市加快发展注入了强大动力。因此，今年的局势挑战是严峻的，困难是空前的，但机遇也是空前的，必须敢于迎头赶上，向低潮要高潮，向危机要机遇，实现"弯道超车"。同时，对许多不确定因素不容麻痹大意，必须审慎谋划，认真应对，励精图治，确保经济社会平稳、较快、持续发展。因此，我们对做好今年各项工作，实现全年奋斗目标充满信心。

　　今年工作的总体要求是：高举中国特色社会主义伟大旗帜，以邓小平理论和"三个代表"重要思想为指导，深入贯彻落实科学发展观，按照省委十届四次全会和市委五届五次全会的部署，围绕"保增长、扩内需、调结构"的总体要求，以学习实践科学发展观活动为动力，以认真实施"砍尾"行动纲要为载体，以抓投入促发展、抓内需促增长、抓改革促创新、抓园区促升级为主线，以切实改善民生为落脚点，以大力优化软环境为抓手，以确保经济社会安全为保障，进一步推进"三大基地"、"三大经济带"建设，实施好"八大工程"，抓住省委、省政府建设海洋强省的机遇，加快发展海岸经济，大力打造港口物流、临港工业、滨海旅游、加工制造、海洋水产等五大龙头产业，推动经济社会又好又快发展，为实现砍尾目标开好局、起好步。

　　今年全市经济社会发展的主要预期目标是：地区生产总值增长16%；全社会工业总产值增长28%，其中规模以上工业总产值增长36%；规模以上工业增加值增长34%；农业总产值增长6%；地方一般预算收入增长25%；全社会固定资产投资增长25%；实际利用外资增长20%；外贸进出口总值增长15%；社会消费品零售总额增长18%；居民消费品价格涨幅控制在3%以内；城镇居民人均可支配收入增长9%，农村居民人均纯收入增长8%；城镇登记失业率控制在3%以内；人口自然增长率控制在6.35&permil;以内；单位生产总值能耗下降3.02%；二氧化硫和化学需氧量分别完成省确定的减排目标。

　　（一）加大重点项目建设力度，以投入拉动保增长

　　加快重点项目建设。继续落实重点项目领导分工责任制。加快推进列入省新十项工程和市重点项目建设，在全市掀起大建设热潮。2009年初步确定市重点建设项目23个，总投资687.9亿元，年度计划投资67.3亿元,预备项目8个，总投资103.5亿元。重点抓好汕尾电厂一期3#、4#机组工程、陆丰核电厂平工程、华润海丰电厂、陆丰甲湖湾新能源基地、市区防洪（潮）工程、华师附中汕尾学校、市区碧桂园商住房工程以及城市、县城污水处理厂等重点项目的建设。贯彻落实中央和省关于扩大内需的政策措施，加强项目的申报和储备，力争多一些项目进入国家、省的计划笼子，力争获得更多投资支持。

　　加快交通基础设施建设。集中力量打响今年交通大会战，加快推进"两网一港"建设步伐。重点抓好国道G324线汕尾段改建工程、汕尾至遮浪公路、省道S242线西闸至埔边段、海丰公平段改线工程等建设。着眼大工业、大港口的衔接配套，完善重大功能性基础设施体系，争取广西玉林至福建漳州高速公路汕尾段动工建设，做好汕尾至江西寻乌高速公路汕尾段前期工作，加快推进厦深铁路汕尾段全线建设，抓紧建设站点配套设施，做好汕尾至龙川铁路、厦深铁路汕尾至广州支线的前期工作。抓紧修编沿海港口总体规划，预留港口、航道发展空间，加大力度推进航道、码头建设。完善口岸各项配套设施，争取扩大汕尾新港红海湾港区开放水域。加快以汕尾新港为龙头的港口群建设，提升大东南出海主通道地位。改造建设陆丰、海丰城东、华侨等汽车客运站和农村客运站点。加快农村公路建设步伐，加大力度建设县通镇、镇通镇、镇通行政村公路，全面完成行政村通水泥路的建设任务。

　　打造宜居宜业新城区。围绕建设宜居宜业滨海新城、粤东重要节点城市的城市定位，推动品清湖沿岸综合开发项目建设，努力提高市区的集聚带动能力。加快推进金湖路、香洲东路等市政道路建设，加快建设金山路、大马路、红海西路、工业园区科技大道等市区主要干道。推进市区东区污水处理厂、西区污水处理厂管道改造工程、城市医疗废物集中处置中心、市区垃圾无害化处理场等市政设施建设，抓紧规划建设市体育中心、民间文化艺术馆等公共设施。加强生态保护，依托原有坡地、山丘，加强城市绿化和园林建设。加大"城中村"改造力度，整合城乡土地，突出扩容提质，拓展城市规模。加强城市环境卫生综合整治，大力营造优美、舒适、卫生的宜居环境，努力把城区建设成为全市政治、经济、教育、文化、消费、医疗中心。

　　（二）加大工业发展力度，做大工业堆头

　　重点发展临港工业、加工制造业，扎实推进"三大基地"建设。

　　打造电力能源产业基地。加快汕尾电厂一期3#、4#机组和陆丰甲湖湾新能源基地建设。开展陆丰核电前期工作，争取国家批准"十一五"规划末期动工建设，争取华润海丰电厂尽快核准，促进风能发电项目尽快建设投产，继续抓好小水电挖潜改造工作。加快推进500千伏茅湖（汕尾）输变电工程收尾工作，抓好220千伏汕尾星云站2#主变扩建工程，以及海丰至公平线路等10个110千伏输变电工程项目建设。

　　打造石油化工产业基地。加快推进上海中石油华城石化储运（生产）基地项目建设。加强推介，吸引更多大型企业落户汕尾发展临港工业。主动承接国际石化产业转移，重点引进一批带动能力强、有利于延伸石化产业链条的项目，打造我市石油化工产业基地，尽快融入粤东石化产业带。

　　打造电子信息产业基地。以信利、德昌电子信息企业为龙头，大力发展以TFT、二极管、三极管等产品为主的电子信息产业。开展电子信息产业集群招商，吸引国内外知名品牌前来投资合作。整合技术、人才、资金、政策、服务等要素，加强产学研合作，把我市电子信息产业融入泛珠三角电子信息产业带，主动承接珠三角电子信息产业转移，形成配套、互补、协调发展的产业链，打造高新技术产业基地。

　　提升壮大传统产业。推动产业结构优化升级，打造优势产业集群。做大做强纺织服装、食品制造、金银珠宝首饰加工和圣诞工艺品等支柱产业。运用高新技术和先进适用技术改造提升传统产业，加快淘汰落后工艺技术，依法有序关闭高污染、高消耗、低效益的企业。

　　（三）着力发展大流通，千方百计扩大内需

　　加快发展港口物流业。依托"一港三铁路三高速"建设的推进，打造全新的物流枢纽。把红海湾港区规划建设成为国际枢纽港口，发展市城区、红海湾开发区现代物流基地；规划建设陆丰湖东煤炭码头，发展辐射粤东北乃至闽赣地区的煤炭转运配送基地；把甲子港、碣石港作为对台小额贸易港口纳入规划，打造陆丰内湖粤东农产品配送基地。

　　加快发展现代服务业。积极推进电子商务的发展，大力培植金融、保险、信息、咨询现代服务业。倾力打造城市商务中心区，重点建设购物、商住、旅游消费项目，大力推进廉租房和经济适用房建设，加快建设珠宝、金银首饰、服装等专业市场和大型农产品、水产品批发中心。加强产品质量和食品药品安全监管，构建保障产品质量安全的长效机制。加强市场价格监管。坚决打击各种哄抬物价、扰乱市场秩序的不法行为。

　　进一步扩大消费需求。拓宽城乡居民收入渠道，创造条件让更多群众增加财产性收入，提升居民消费能力，增强消费对经济增长的拉动力。拓展农村消费市场，推进"万村千乡"市场工程和农资配送中心建设，促进农民消费。积极开拓国内市场，扩大消费需求。培育旅游、文化、体育、保健、信息服务等消费热点，推动消费结构升级。加强消费者权益保护，优化消费环境，增强消费信心。

　　加快发展特色旅游经济带。突出抓好旅游发展规划，加大旅游招商力度，促进旅游产业上新台阶。重点整合"红、蓝、绿、古"四色资源，构建不同风格的滨海、生态和城市景观区。着力打造市区城市游憩旅游区；红海湾海洋运动区；鲘门滨海休闲度假区；碣石玄武山、金厢滩宗教海滨旅游区、赤坑温泉度假区、陆河绿色生态旅游区。完善旅游基础设施，建设高星级酒店，丰富旅游内涵，拓展旅游空间。加强旅游促销，不断开拓旅游市场。加强与粤、闽、赣旅游资源的整合与协作，吸引更多国内外游客来汕尾观光旅游。

　　（四）强力推进招商引资，实现内外源经济协调发展

　　抓紧修编有关规划。高度重视产业布局规划和城市总体规划，编制海岸经济产业规划，明确产业功能定位和产业发展方向。切实做好产业规划与城乡总体规划、土地利用规划、主体功能区规划、环境规划的衔接工作，促进生产要素有序流动和产业合理布局。提高城市控制性详细规划覆盖率，发挥规划引领发展的龙头作用，大力推进海岸经济的发展。

　　突出抓好园区建设。对现有工业园区进行优化组合，构筑工业发展大平台。继续推进红海湾经济开发区、海丰老区经济开发区、陆丰东海经济开发区、星都经济开发区等省级开发区的建设。尽快申报设立保税仓、快件中心、出口加工区等特殊监管区。继续推进"双转移"工作，加强与深圳等珠三角地区的合作交流，通过产业转移园区招商，促进在产业、资金、资源、人才、劳动力等方面的优势互补。重点做好深圳（汕尾）产业转移工业园的开发工作，逐一落实好入园项目，争取尽快建成省级产业转移示范区，全力建设好园区投资环境。

　　加大招商引资力度。继续落实招商引资责任制，创新招商引资方式，拓宽招商引资渠道，优化利用外资结构，切实提高利用外资质量和水平。运用"借用外脑、借用外力、借用外财"的策略，采取融进来、接过来、借进来的办法，在智力、产业、招商、物流、资金等方面广泛建立战略合作伙伴关系，加强与大财团、大企业和高等院校等的合作。加大对外推介和宣传，认真组织参加各种境内外经贸活动，在工业、服务业、物流业、城市经营、土地及港口资源开发等方面进行全方位招商合作。

　　优化投资环境。实施"共克时艰，抱团取暖"策略，优化企业生产经营环境，共担风险度过难关。继续推进"大通关"建设，不断改善投资营商环境。提高服务质量，完善优惠政策，推进市行政服务中心建设，进一步提高项目审批和服务水平，建立和完善集中联合审批制度、新投产项目自主研发奖励制度、"金钥匙"保姆式项目服务制度、领导干部招商引资责任制等配套措施，为投资者提供优质高效的服务。创新园区管理机制，形成责任、效能、廉洁管理机构。

　　扩大外贸出口。用足用好中央、省一系列保障出口政策，大力支持优势企业和产品扩大出口。加强政策扶持、市场引导和行业服务，切实解决企业面临的困难和问题，稳定加工贸易，扩大一般贸易，促进加工贸易转型升级。鼓励民营企业发展加工贸易，推进加工贸易企业开拓国内市场，扩大内销。重点做好信利、德昌、万盛、敏兴等一批大型企业的服务工作。加快出口商品生产基地建设，提升一般贸易出口水平。

　　推进内源型经济上新台阶。扶持促进中小企业发展，引导企业准确把握国内市场需求，调整优化产品结构，抢占国内市场。组织参加"山洽会"、"中博会"、"侨博会"等经贸活动。实施名牌带动战略，鼓励支持重点企业争创全国、全省名牌产品和著名商标，培育和发展一批区域品牌。推动民营经济发展，优化提升专业镇水平，开展"千商回乡、百企入汕"活动，形成全民创业高潮。

　　（五）大力发展现代农业，促进绿色、蓝色经济带发展

　　加快发展效益农业。打造农业种养"十大基地"、建设"五大园区"。培育发展一批国家、省、市级农业龙头企业和专业合作社组织。全面推广"公司+基地+农户"经营方式，不断增加农民收入。加快市现化农业示范园区建设，提升全市农业现代化水平。

　　加快发展海洋水产业。大力发展海洋捕捞业和水产品深加工业，积极推广养殖新技术，提高海淡水养殖水平。加快水产养殖基地化建设，重点建设罗非鱼、对虾、牡蛎、鲍鱼、海马等海产品养殖基地，以及深水抗风浪网箱养殖基地，努力打造海洋经济支柱产业。加强水生野生动物的保护管理以及贝类等海产资源保护区的管理，加快人工鱼礁区的建设。稳步推进捕捞渔民转产转业，大力创办水产养殖专业合作社。加强渔船渔场渔港和水产品市场的管理，完善水产品质量安全管理体系。

　　加强农田水利设施建设。启动"百里海堤爱海工程"，分2至3年建设加固2050年一遇标准的240公里海堤，维护海岸生态，爱护海岸经济。按照"路相通、渠相连、林成网、田成方、涝能排、旱能灌、地力高、生态好"的标准，加强农田基础设施建设。重点建设各县（市、区）防洪工程，继续抓好省人大水利议案工程的实施，加快防灾减灾、饮水安全、小型水库除险加固、农村机电排灌等工程建设。

　　拓宽农民增收渠道。大力发展壮大县域、镇域经济，千方百计增加农民收入。开展科技进村入户服务活动，努力把广大农户培养成有较强市场意识、有较高生产技能、有一定管理能力的现代农业经营者，促进农业增产农民增收。发展品牌和集约经营，带动更多农户增加收入。

　　大力改善农村生产生活条件。加大强农惠农力度，落实粮食最低收购价、对种粮农民增加补贴等惠农政策，改善农业生产条件，减轻农民负担。增加农业投入，加强农村能源、交通、人畜饮水和农产品质量安全等基础设施建设。整合各级政府资源以及社会资源，促使农村饮水安全工程实现根本性突破。继续抓好贫困户危房改造工程。认真抓好农村环保工作。全面做好农村土地延包工作，引导农民以多种形式流转土地承包经营权，加强被征地农民的社会保障。

　　（六）加强管理体制改革，提高融资理财管理水平和抗风险能力

　　加强税收征管。做大做强支柱产业，大力拓宽财源税源。坚持依法治税，加强税收宣传，优化纳税服务，适度优化税赋比例结构，提高依法纳税遵从度。坚持和完善领导责任分工、财税工作分析和联席会议等工作机制，密切部门配合，充分发挥党政职能部门护税协税整体效能作用。加强重点税种、重点税源企业和重点项目管理，确保收入快速增长。加大税务稽查力度，严厉打击各种偷骗税行为，整顿和规范税收秩序。加快建立国有资本经营预算制度，规范政府资源有偿使用收入管理。

　　推进财税改革。深化预算制度改革，强化预算管理和监督，推进国库集中收付、财务核算集中监管等改革。提高理财能力，充分发挥财政专项资金"四两拔千斤"作用，运用金融手段提高政府财力和办实事能力。完善财政激励机制和配套措施，调动县级特别是镇级发展县域镇域经济的积极性和主动性，积极解决镇级财政困难，不断提高县（市、区）和镇级财政保障水平。

　　加强国有资产管理体制改革。向改革要活力。以盘活旧资源、创建新机制、激发新活力、增添新价值为指导原则，引导国有企业面向市场进行改制重组，逐步化解历史包袱，理顺国有资产管理体制机制。使经营不善者走出困境，使优质资产整合升级，使劣势资产及时消化。逐步创造条件组建上市公司。

　　强化公共财政服务功能。落实配套中央、省扩大内需财政扶持政策，整合财政性引导资金，扶持一批重点项目开工建设，早日发挥效益。发挥公共财政的功能作用，注重以民生为重点的社会事业建设，加大对社会公共服务配套设施的投入，不断优化支出结构。实施城乡收入倍增计划。大力提倡节支增收，加强对"三农"、教育、卫生、文化、基础设施、社会保障、维护稳定等的投入，提高财政资金效益。

　　防范化解财政和地方金融风险。依法规范政府投融资行为，制订完善政府举债和担保制度，尽快建立担保公司和中介机构，构建中小企业融资平台，进一步扭转信贷比被动状况。加大对民生工程的贷款投入，优化金融生态环境，切实采取措施，防范和化解银行业风险，督促银行业继续做好不良贷款清收处置，维护财政金融安全。

　　（七）大力加强社会保障，推进社会事业协调发展

　　强化社会保障工作。大力推进社会保险扩面征缴，加快推进城镇居民基本医疗保险制度，认真解决困难企业退休人员参加医疗保险问题，落实好军队退役人员劳动保障政策。加强老龄工作，确保企业离退休人员养老金等社保待遇按时足额发放。完善城乡居民最低生活保障制度，做好低保、农村五保供养和孤儿救助工作，逐步提高保障水平。做好优抚安置工作。坚持实施积极的就业政策，加强政府引导，完善市场就业机制。落实省劳动培训转移政策，分类组织培训，把实施"百万工程"与农民工技能提升培训工作有机结合起来，实现转移就业，全市培训农村劳动力1.8万人，转移农村劳动力3.8万人。以创业带动就业，扩大就业规模。继续实施扩大与促进就业工程，重点帮助下岗失业人员、零就业家庭、残疾人、农村贫困家庭、被征地农民和转产转业渔民就业。

　　优先发展教育事业。实行"议教"制度，逐年解决教育遗留问题，突破发展的重要瓶颈。以大力提升高中、初中、小学入学率、就学率、升学率为主线，大力加强教育工作。加强教育城域网建设，推进教育现代化，重视学前教育，关心特殊教育，鼓励和规范民办教育，发展远程教育和继续教育。大力发展职业技术教育和职业技能培训，建设好高技能人才公共实训基地，加快技工学校扩建工程的完善配套。全面实行免费义务教育，增强高中自主办学能力，进一步加快高中阶段教育发展步伐，推进示范性普通高中建设。加快推进华师附中汕尾学校上马建设。加强师德师风师能建设，切实提高教师素质，大力推进中小学代课教师待遇问题及时解决。

　　全面发展社会事业。加强农村三级卫生服务网络和城市社区卫生服务中心建设，加强农村医疗人才队伍建设，完善城乡卫生服务体系。深入开展爱国卫生运动，发展妇幼卫生事业。提高新型农村合作医疗管理和保障水平，提高人均补助标准。发展社会福利和慈善事业。广泛开展全民健身和竞技体育运动，不断提高全民体育健身意识。加快推进2010年亚运会汕尾赛区筹备工作。创新人口与计划生育管理机制，深入开展"谁英雄谁好汉，'两无'活动比比看"活动，落实层级动态管理责任制，提升人口与计划生育工作水平。大力推行火葬，扭转殡改工作被动局面。完善救助管理机制。认真实施妇女儿童发展规划，加强对未成年人和妇女的权益保护。加强军政军民团结，做好双拥工作。加强国防教育和国家安全教育，进一步提高民兵预备役建设水平。促进统计、人防、气象、防震、台务、港澳、老龄、文化广播电视、新闻出版、民族宗教、外事侨务、档案修志等工作全面发展。

　　2009年要重点办好十件民生实事：1、全面启动解决全市农村60万农民饮水安全问题工程。2、春节前后解决市城区马宫街道居民饮水问题。3、大力推进廉租房建设，2009年完成450套。4、提高社会保险市级统筹层次，实行全市社会保障城乡覆盖一体化。5、开工建设市民间文化艺术馆。6、建设市儿童福利院。7、建设市妇幼保健院。8、开工建设华师附中汕尾学校、汕尾理工职中。9、建设红海西路，推进西区排污管网建设并解决相关区、街浸水问题。10、改造大马路1100米。

　　（八）打造发展软环境，树立汕尾新形象

　　打造法治环境。切实加强民主政治建设，完善人大代表、政协委员反映民意的机制，严格按照法定权限和程序履行职责。认真落实市人大及其常委会各项决议决定，坚持向人大及其常委会报告工作，主动加强同政协的联系并向其通报工作，自觉接受监督。提高议案、建议和提案办理质量。坚持原则，依法办事，切实维护人民群众的利益。完善民主党派、无党派人士建言献策"直通车"制度。积极推进政务公开、村务公开、厂务公开，扩大基层民主。加快推进依法治市进程，加强行政机关、司法队伍权威性建设，坚决落实依法行政、公正司法和严格执法。加强法制宣传教育，提高公民的法律素质。

　　打造平安环境。强化"人为本、和为贵、教为先、法为上、久为功"的理念，大力构建全党动员、全民参与、全员激励的大平安体系。切实健全应急管理机制，提高预防和处置突发事件能力，最大限度减少突发事件发生。认真开展各种严打专项整治，依法防范和打击违法犯罪活动，坚决遏制"黄赌毒"，构建反走私打防体系。加强劳动保障监察，依法维护劳动权益。切实抓好信访和社会调解工作，严格坚持排查调解工作制度，完善大接访长效机制；建立社会投诉受理化解新机制，及时将社会矛盾纠纷化解在萌芽状态。坚持安全发展。全面加强产品质量安全工作，强化对涉及人民健康和安全的食品、药品等重点产品的监管，构建保障产品质量安全的长效机制。扎实推进各项专项整治，坚决防范和遏制重特大安全事故发生。加强消防、道路交通、水上交通安全工作。做好防灾减灾工作。

　　打造创新环境。营造"鼓励创新、宽容失误、拒绝平庸"的氛围，出台鼓励创新优惠政策，完善自主创新激励机制。做好人才引进工作，打造"创新乐土、人才洼地"。落实科技三项经费的投入，加快省、市专业技术创新试点镇建设。建立健全区域技术创新和特色产业基地技术创新服务平台，为中小企业提供科技服务和技术支持。引导企业在加强引进的同时，加大对科研、技术的投入力度，改造、提升和开发新的技术设备，提高原始创新和引进消化吸收再创新能力，促进产品由低端向高端提升，不断增强企业和产品竞争力。鼓励企业加强与高等院校和科研院所的产学研合作，加快科技成果的转化与应用。

　　打造政务环境。大力推进"形象年"实践活动，建设"权威、责任、效能、廉洁"政府。坚持以"忠"生威，强化政治责任；以"法"生威，矢志依法行政；以"信"生威，恪守政府信用；以"情"生威，密切群众关系；以"勤"生威，树立勤政形象；以"能"生威，增强执政能力；以"严"生威，维护法治尊严；以"廉"生威，保持清正廉明，建设权威政府。进一步完善岗位目标责任制、首问责任制、服务承诺制和限时办结制等制度，把落实责任贯穿于政府工作的全过程，全面贯彻《广东省市厅级党政领导班子和领导干部落实科学发展观评价指标体系及评价办法》，进一步建立和完善对市直单位和县（市、区）的考核体系，加强检查督促，严肃查处行政不作为和乱作为，建设责任政府。深化行政审批制度改革，简化审批程序，规范审批行为，提高行政审批效率。推进电子政务建设，及时把政府决策、服务程序、办事方法向社会公布。健全和完善行政审批电子监察系统。建立和完善重大问题集体决策制度，提高决策的透明度和公众参与度。强力提高行政效率，建设效能政府。认真落实反腐倡廉建设各项规定。加强对领导干部、人财物管理使用关键岗位的监督和制约，强化行政监察，严防失职渎职等违法犯罪现象发生。完善政府采购、项目招投标等制度，堵塞漏洞。加强机关作风建设，加强对公务员的教育、培训和管理，努力建设一支政治坚定、业务精通、勤政廉洁、作风优良的公务员队伍，建设廉洁政府。

　　打造生态环境。贯彻"穷人也要挑菜吃"理念，认真落实节能减排责任制，严格环保责任考核制度。加强对大湖鸟岛、公平自然生态保护区、陆河红椎林保护区的保护；加强造林绿化、水土保护、环境保护工作，重点实施市区周边山地造林绿化工程，落实森林防火责任制和责任追究制，为崛起蓝色汕尾创造相适应的绿色环境。实行工业园区建设与生态环境控制同步规划、同步建设、同步运营，大力发展循环经济，推行清洁生产，广泛开展节能节水节地节材活动，促进节能增效。严格控制建设占地规模，严格查处违规用地，进一步盘活存量土地，重点消化利用各类闲置土地，提高土地利用效率。严格环保市场准入，从源头上控制污染。

　　打造人文环境。加强社会主义核心价值体系建设，大力弘扬民族精神、时代精神和新时期汕尾人精神。把彭湃同志"敢为人先、无私奉献"精神转化为全市干部群众解放思想、锐意进取的优良作风；把"中国民间文化艺术之乡"的浓厚底蕴转化为礼仪儒雅的教化优势；动员社会各方面共同做好青年学生思想政治教育和未成年人思想道德教育工作。推行科学文明健康的生活方式。广泛开展社会公德、职业道德、家庭美德教育活动，促进群众性精神文明创建活动上新水平。推进文化体制机制创新。加强文化基础设施建设，完善文化公共服务体系，弘扬先进文化，繁荣群众文化，发掘和研究传统优秀文化，打造独具海陆丰特色的文化品牌。加强文化遗产和优秀民俗民间文化的保护和开发利用。创造条件举办全国性的民间文化艺术交流活动，提升"中国民间文化艺术之乡"的影响力。以庆祝新中国成立60周年系列文化活动为主线，努力繁荣文艺创作，推出艺术精品，举办群众性地方戏剧文化活动，继续抓好以"欢乐广场，唱响汕尾"为主题的群众性广场文化活动。加强对外文化交流，增强海陆丰文化的开放性、兼容性、时代性，激发全民文化创造活力，提高文化软实力。

　　各位代表，同志们，新的使命考验我们，新的目标催人奋进。让我们更加紧密地团结在以胡锦涛同志为总书记的党中央周围，高举中国特色社会主义伟大旗帜，以邓小平理论和"三个代表"重要思想为指导，深入贯彻落实科学发展观，在市委的坚强领导下，同心同德，开拓创新，扎实工作，为把汕尾建设成为工业、旅游业和海洋产业发达的现代化滨海新城而努力奋斗！
